# Non‐bacterial thrombotic endocarditis with cryptogenic stroke

**DOI:** 10.1002/ccr3.4833

**Published:** 2021-09-15

**Authors:** Hirokazu Toyoshima, Bun Nakanura, Motoaki Tanigawa, Hiroyuki Tanaka, Yuki Nakanishi, Shigetoshi Sakabe

**Affiliations:** ^1^ Department of Infectious Diseases Japanese Red Cross Ise Hospital Ise Japan; ^2^ Department of Thoracic and Cardiovascular Surgery Mie University Tsu Japan; ^3^ Department of Respiratory Medicine Japanese Red Cross Ise Hospital Ise Japan

**Keywords:** cryptogenic stroke, lifelong anticoagulation, non‐bacterial thrombotic endocarditis, surgery, transesophageal echocardiography

## Abstract

Negative blood culture and pathological findings are helpful to diagnose non‐bacterial thrombotic endocarditis. The treatment strategy, including lifelong anticoagulation or surgery, should be individualized based on patients' underlying diseases.

## Graphical abstract

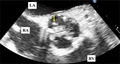

Polypoid lesions in the aortic valve are common in the clinical setting, with infective endocarditis as the most common cause. However, its differential diagnoses include non‐bacterial thrombotic endocarditis, such as Libman‐Sacks endocarditis, Lambl's excrescences, and papillary fibroelastoma. Thus, careful diagnosis is crucial, and the management needs to be individualized.

An 83‐year‐old Japanese woman with diabetes presented with dyspnea on exertion due to severe aortic stenosis and a polypoid lesion on transesophageal echocardiography (Figure 1). She was prescribed an anticoagulant for recurrent strokes. Physical examination revealed moderate systolic murmur without fever. Laboratory findings were as follows: white blood count, 5700/µl with 69.0% neutrophils; C‐reactive protein, 0.56 mg/dl; hemoglobin A1c, 6.4%; and antinuclear antibody, negative. Three sets of blood cultures were negative. The patient underwent surgical aortic valve replacement, and a 1 × 5‐mm polypoid structure was removed. Pathological examination revealed thrombus, calcification, and fibrous tissue (Figure 2); Elastica van Gieson staining results and sample culture were negative. The patient was diagnosed with non‐bacterial thrombotic endocarditis (NBTE) but remains disease‐free without stroke or recurrence.

**FIGURE 1:**
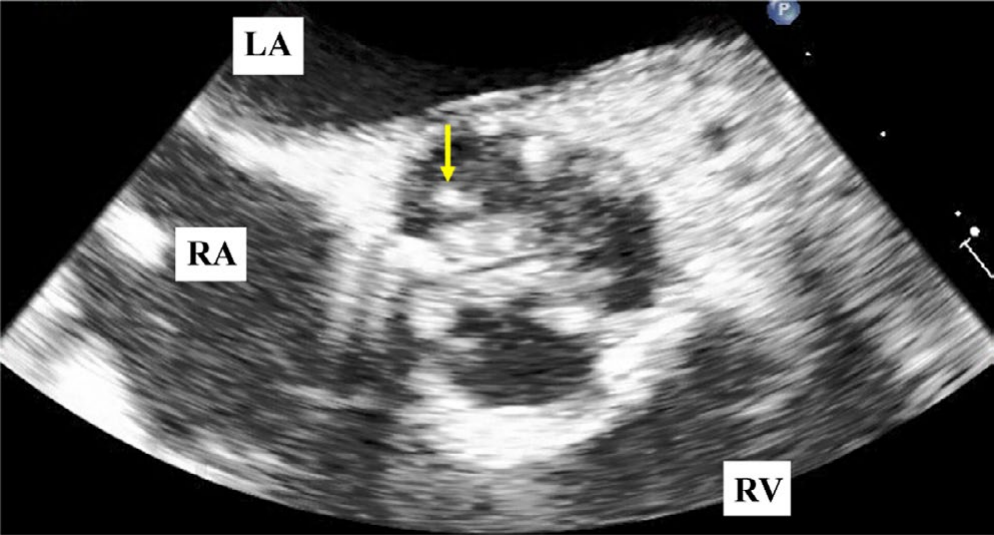
A polypoid lesion on the non‐coronary cusp (yellow arrow) with a calcified aortic valve on transesophageal echocardiography. LA, left atrium; RA, right atrium; RV, right ventricle

**FIGURE 2:**
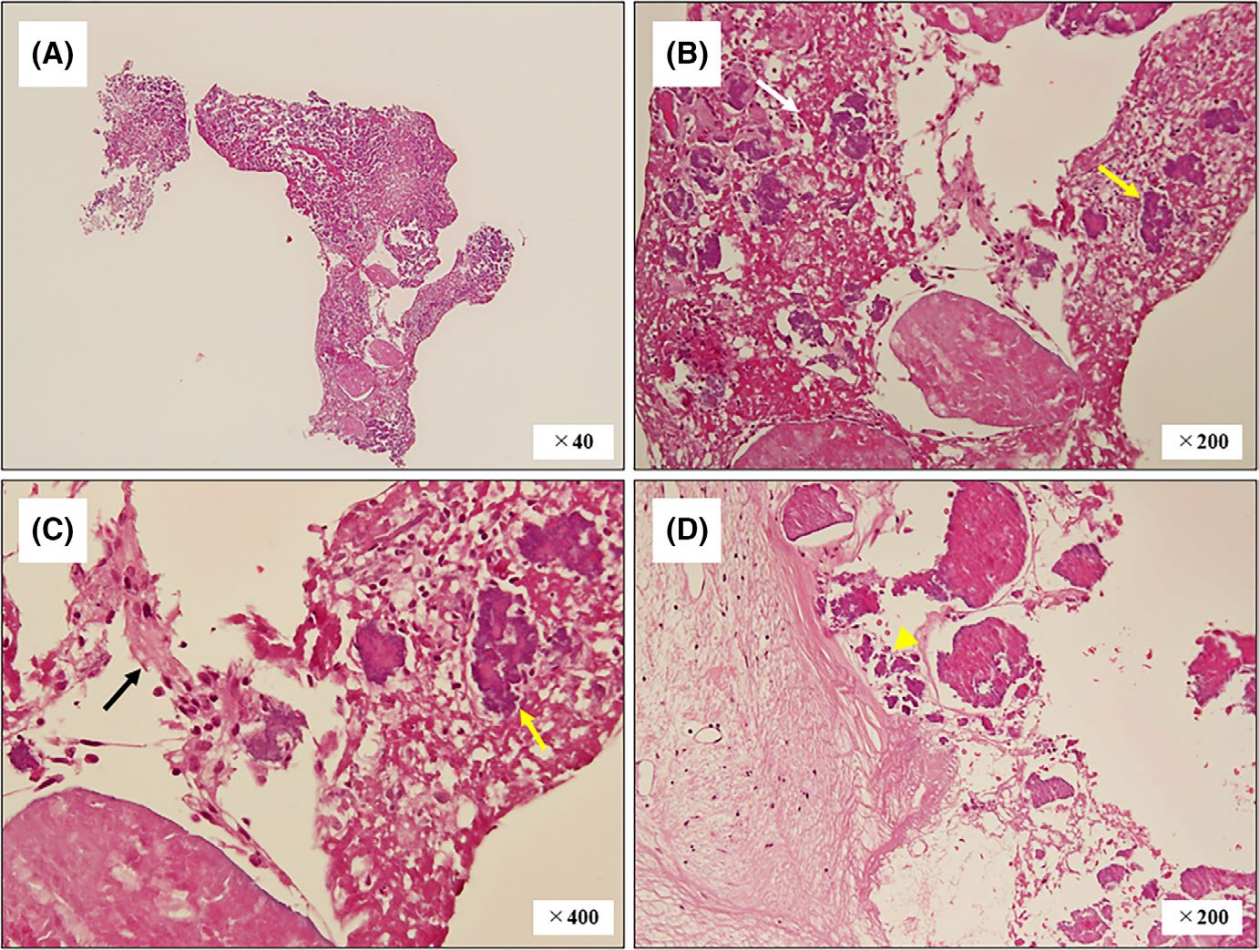
The sample findings of hematoxylin and eosin staining (A–C). The sample consisted of an acidophilic amorphous structure (B, white arrow), suggesting thrombus and a slight amount of fibrous tissue (C, black arrow) with calcification (B, C, yellow arrows). Additionally, calcification of the aortic valve without inflammation was also seen (D, yellow arrowhead). These excluded Lambl's excrescences and papillary fibroelastoma and suggested non‐bacterial thrombotic endocarditis with calcification

Non‐bacterial thrombotic endocarditis is a type of noninfective endocarditis commonly associated with pre‐existing comorbidities like diabetes (40%) and malignancy (59%), especially in patients over 70 years.^1^ Negative blood culture and pathological findings are important to diagnose NBTE because patients with infective endocarditis can also be apyrexial.^2^ Anticoagulation may decrease stroke risk; however, surgery should be considered in severe valvular disease cases or recurrent strokes despite anticoagulation.^2^ NBTE treatment involves lifelong anticoagulation or surgery and should be individualized based on patients' underlying diseases.

## CONFLICT OF INTEREST

None declared.

## AUTHOR CONTRIBUTIONS

HToyoshima contributed to the clinical management of the patient, was involved in study conception, acquisition and analysis of the data, and drafting of the manuscript. BN contributed to the clinical management of the patient. HTanaka and YN involved in study conception. TM and SS involved in supervision of the drafting of the manuscript and critical revision of the manuscript. All authors reviewed the final draft of the manuscript and approved its submission.

## ETHICAL APPROVAL

This study has been approved by the institutional review board and ethics committee of Japanese Red Cross Ise Hospital (Permission number: ER2021‐7).

## CONSENT

Written informed consent was obtained from the patient for publication of this case report and the accompanying images. A copy of the written consent is available for review from the editor in chief of this journal on request.

## Data Availability

The data that support the findings of this study are openly available in [repository name e.g “figshare”] at http://doi.org/[10.1002/ccr3.4833], reference number [CCR3‐2021‐04‐0695‐IV.R1].
